# Impact of electronic medication reconciliation interventions on medication discrepancies at hospital transitions: a systematic review and meta-analysis

**DOI:** 10.1186/s12911-016-0353-9

**Published:** 2016-08-22

**Authors:** Alemayehu B. Mekonnen, Tamrat B. Abebe, Andrew J. McLachlan, Jo-anne E. Brien

**Affiliations:** 1 Faculty of Pharmacy, University of Sydney, Sydney, Australia; 2 School of Pharmacy, University of Gondar, Gondar, Ethiopia; 3 Centre for Education and Research on Ageing, Concord Hospital, Sydney, Australia; 4 St Vincent’s Hospital Clinical School, Faculty of Medicine, University of New South Wales, Sydney, Australia

**Keywords:** Electronic medication reconciliation, Medication history, Medication safety, Medication errors, Medication discrepancies, Care transition

## Abstract

**Background:**

Medication reconciliation has been identified as an important intervention to minimize the incidence of unintentional medication discrepancies at transitions in care. However, there is a lack of evidence for the impact of information technology on the rate and incidence of medication discrepancies identified during care transitions. This systematic review was thus, aimed to evaluate the impact of electronic medication reconciliation interventions on the occurrence of medication discrepancies at hospital transitions.

**Methods:**

Systematic literature searches were performed in MEDLINE, PubMed, CINHAL, and EMBASE from inception to November, 2015. We included published studies in English that evaluated the effect of information technology on the incidence and rate of medication discrepancies compared with usual care. Cochrane’s tools were used for assessment of the quality of included studies. We performed meta-analyses using random-effects models.

**Results:**

Ten studies met our inclusion criteria; of which only one was a randomized controlled trial. Interventions were carried out at various hospital transitions (admission, 5; discharge, 2 and multiple transitions, 3 studies). Meta-analysis showed a significant reduction of 45 % in the proportion of medications with unintentional discrepancies after the use of electronic medication reconciliation (RR 0.55; 95 % CI 0.51 to 0.58). However, there was no significant reduction in either the proportion of patients with medication discrepancies or the mean number of discrepancies per patient. Drug omissions were the most common types of unintended discrepancies, and with an electronic tool a significant but heterogeneously distributed reduction of omission errors over the total number of medications reconciled have been observed (RR 0.20; 95 % CI 0.06 to 0.66). The clinical impact of unintended discrepancies was evaluated in five studies, and there was no potentially fatal error identified and most errors were minor in severity.

**Conclusion:**

Medication reconciliation supported by an electronic tool was able to minimize the incidence of medications with unintended discrepancy, mainly drug omissions. But, this did not consistently reduce other process outcomes, although there was a lack of rigorous design to conform these results.

**Electronic supplementary material:**

The online version of this article (doi:10.1186/s12911-016-0353-9) contains supplementary material, which is available to authorized users.

## Background

Medication reconciliation has been recognized as an important approach to improve the quality use of medicines by reducing the burden of medication discrepancies at care transitions [1–4]. Medication discrepancies often occur at transitions in care when patients are admitted to and discharged from a hospital, and are responsible for more than half of the medication errors [5]. Unintentional medication discrepancies are highly prevalent at hospital transitions — for example, two-thirds of inpatients have at least 1 unexplained changes to medication at hospital admission [6], and up to one-third of the medication discrepancies could have a potential for patient harm [7]. Clinically important medication discrepancies could also represent an important cause of adverse drug events (ADEs) [8–10] and healthcare resource utilization [11, 12] during transitions in care.

Medication reconciliation has been adopted and championed by a number of patient safety organizations. Medication reconciliation (MedRec) is defined by the Institute for Healthcare Improvement (IHI) as “the process of identifying the most accurate list of a patient’s current medicines including the name, dosage, frequency and route — and comparing them to the current list in use, recognizing and documenting any discrepancies, thus resulting in a complete list of medications” [13]. Depending on the resources available, various approaches to the medication reconciliation intervention are employed internationally, including the use of electronic reconciliation tools [14–16], standardized forms [17, 18], collaborative models [19, 20], and pharmacy-led programs [21, 22]. Particularly, the use of information technology (IT) can increase the accuracy of documentation used for the medication reconciliation, and is now commonly used to facilitate the reconciliation process [23]. One of the main advantages of the electronic medication reconciliation is that the best medication history can be ensured through information sharing [24]. IT-related interventions might reduce medication discrepancies at hospital transitions [25], but there are fewer studies supporting this evidence. Additionally, previous reviews [26, 27] included medication reconciliation interventions carried out by physicians, nurses, pharmacists and electronic medication reconciliation and evaluated both clinical (e.g. hospital readmissions) and process outcomes (e.g. medication discrepancies), but did not specifically assess the impact of electronic medication reconciliation. In the literature, numerous reviews [28–30] examined the impact of electronic prescribing on medication errors and ADEs; however, no reviews have yet examined the impact of IT on medication discrepancies identified through the medication reconciliation process. The purpose of this study was thus, to systematically evaluate the available literature on the effectiveness of electronic medication reconciliation in reducing medication discrepancies during transitions in hospital care.

## Methods

### Search strategy

This systematic review and meta-analysis was performed according to the PRISMA statement [31], including a checklist to ensure consistent reporting of a systematic review. The search included articles from inception of the databases up to week 3 of November 2015, which were obtained through an extensive search of the following electronic databases: MEDLINE (1946), EMBASE (1966), CINAHL (1937) and PubMed (1946). Some of the key words or Medical Subject Heading (MeSH) terms used in the search were: “medication reconciliation,” “medication discrepancies,” “medication errors,” “medication history,” “electronic health records,” “patient admission,” “patient discharge,” “patient transfer,” and “hospital”. Details on the specific search terms and combinations are provided in the Additional file 1. The literature search also involved manual search of bibliographies of the identified papers. Only studies published in English were included. No restrictions were imposed on year of publication.

### Study selection

Two independent reviewers (ABM, TBA) screened abstracts and titles for eligibility. When the reviewers felt that the abstract or title was potentially useful, full copies of the articles were retrieved and considered for eligibility by the reviewers. When discrepancies occurred between reviewers, the final decision was made based on the agreement of these reviewers.

To be included in the selection, studies required to present all of the following: studies which reported data related to the effectiveness of electronic medication reconciliation intervention, and provided data on medication discrepancies or errors. Medication discrepancies were defined as one or more differences in (dosage, frequency, drug, and route of administration), as described by the IHI [13], between the current and previous medication (s) a patient was taking. We excluded studies with a focus on other types of medication errors (e.g. prescribing errors) that were identified through the non-reconciliation process. The included interventions had to start in the hospital and must be performed primarily by an electronic tool with the aim of minimizing medication discrepancies during transitions in hospital care. Regardless of the study design, the intervention must be compared with another group that received usual or standard care. ‘Usual care’ was defined as any care in which medication reconciliation was not supported by an electronic tool, or if there was not any previous formal electronic medication reconciliation in place. Only full-text published articles from peer-reviewed journals were eligible for inclusion. Along with duplicate references and studies with a different focus, the following types of studies were excluded: other medication reconciliation practices (e.g. pharmacist-led medication reconciliation programs not supported by technology), case studies, systematic reviews, qualitative outcomes, and non-research articles. Abstracts from conferences and full-texts without raw data available for retrieval were not considered.

### Data extraction and quality assessment

Two study authors (ABM, TBA) independently extracted data in a standardized form, including quality assessment of randomized studies [32]. Observational studies were evaluated for their quality by applying criteria from the ACROBAT-NRSI statement including: 1) bias due to confounding, 2) bias in selection of participants into the study, 3) bias in measurement of interventions, 4) bias due to departures from intended interventions, 5) bias due to missing data, 6) bias in measurement of outcomes, and 7) bias in selection of reported results [33]. The response for each criterion was judged based on a scale of low, moderate, serious, critical and no information. Any disagreements between the authors were resolved with mutual consensus. In general, we abstracted the following data: author, year of study, country of origin, study setting and design, number of study participants, target of transition, description of the intervention, length of the study, medications assessed for discrepancy and whether those discrepancies were explicitly described as unintentional changes to medications after clarification was sought from the medical team and/or patient. The primary outcome of interest was the rate and incidence of medication discrepancies, expressed in terms of the proportion of patients with medication discrepancies, or as a mean number of discrepancies per patient, or the proportion of medications/medication orders with discrepancies over the total number of medications reconciled. The secondary outcome was an assessment of the clinical relevance of identified medication discrepancies.

### Statistical analysis

Meta-analyses of studies were done according to the *Cochrane Handbook for Systematic Review of Interventions* [34], using the Review Manager (RevMan) Version 5.3. (Copenhagen: The Nordic Cochrane Centre, the Cochrane Collaboration, 2014. (http://tech.cochrane.org/revman/). A random-effects model was employed, and the results were presented in forest plots. For studies providing dichotomous data, the relative risk (RR) with its 95 % confidence interval (CI) was calculated by comparing medication discrepancy rates between the intervention and comparison group. Whereas for continuous data, we calculated the mean differences with their associated 95 % confidence intervals. We assessed statistical heterogeneity by observing τ^2^, *χ*^2^ (Q), I^2^ and *p*-value. We attempted to explore the possible sources of heterogeneity through subgroup analysis; however, the inclusion of too few studies in each of the outcomes studied precluded us from carrying out such analyses. Sensitivity analysis was carried out to assess the stability of pooled estimates when any of the studies were withdrawn from the analysis. *P*-value < 0.05 was considered as statistically significant. Publication bias was not assessed with funnel plots because the number of studies included in the meta-analyses were too few in this report.

## Results

### Search results

**Fig. 1:**
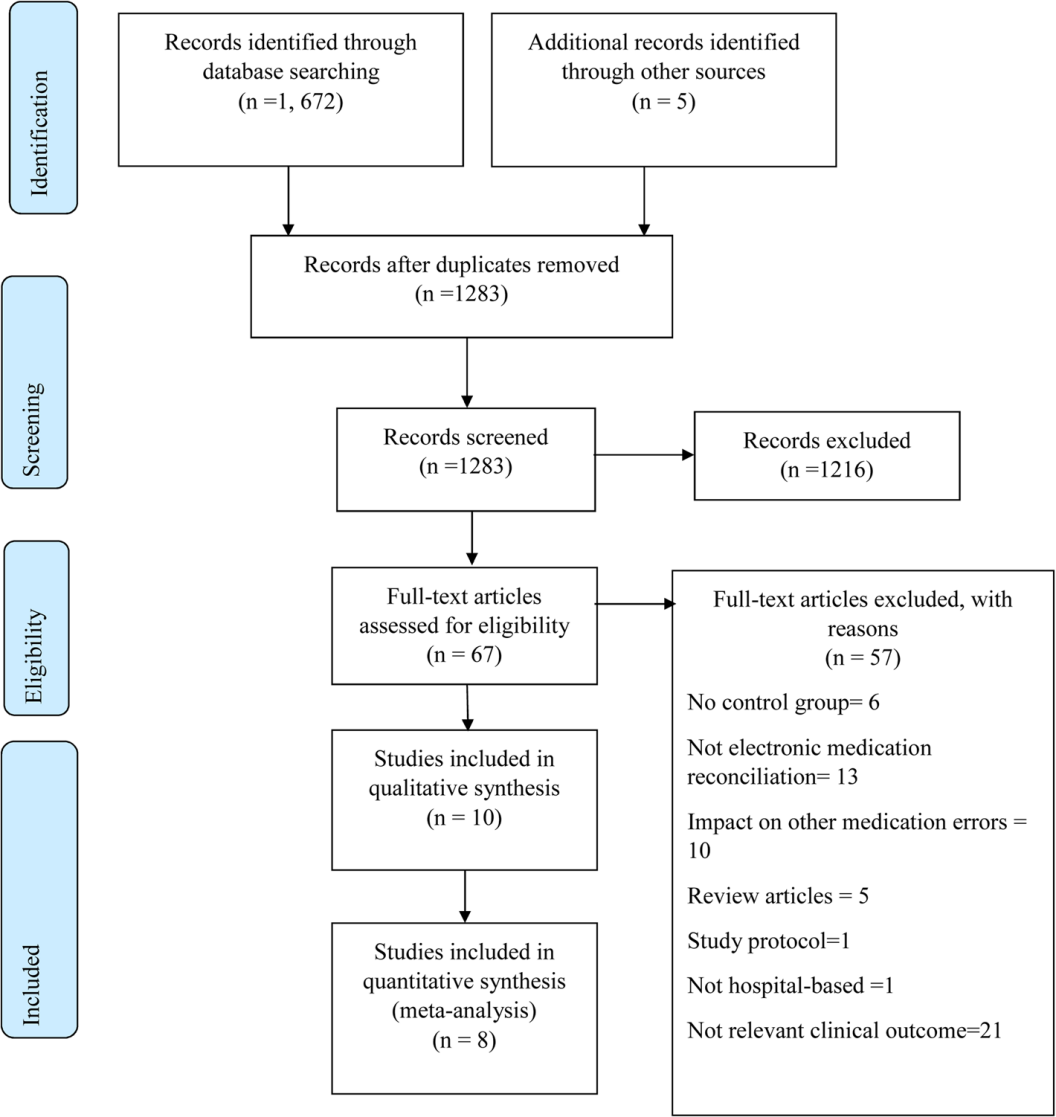
PRISMA flow diagram of included studies

The initial electronic database search resulted in a total of 1672 articles. An additional 5 studies were identified through hand-search of the reference lists of included studies. On removal of duplicate records, 1283 studies were screened for title and abstract. Of these, 1216 studies did not meet the selection criteria. Of the 67 studies obtained in full-text, only 10 studies met the inclusion criteria (Fig. 1). The main reasons for exclusion were either due to reporting of a different outcome of interest (*n* = 21) or medication reconciliation was not supported by information technology (*n* = 12) (Additional file 2).

### Characteristics and quality of included studies

#### Study characteristics

Detail characteristics of the included studies are summarized in Table 1. The included studies were published between 2006 and 2015, and entirely performed in the USA [35–41] and Spain [42–44]. Only one study [40] was a randomized controlled trial. The remainder were non-randomized studies, mainly employing a pre-post study design. All except one study [41] were performed in academic centres or tertiary care hospitals. Two studies [37, 40] were conducted at multiple centres. Nine of the 10 included studies involved a total of 21,486 patients of various sample sizes ranging from 100 to 19,476 patients/discharges. The length of study periods ranged from 10 to 70 weeks. The included studies were heterogeneous for interventions, outcomes, target of transitions and methods for measuring outcomes. Electronic medication reconciliation interventions were more variable with regard to the place of transition, and commenced at various points of hospital transition, such as admission [35, 37, 42–44], discharge [36, 41] and multiple transitions [38–40]. Besides the development of an electronic medication reconciliation tool, some studies utilized a multifaceted intervention, including involvement of a computerized reminder alert [35], process re-design (e.g. work-flow) and staff training [40, 44], and integration of an electronic tool with an already existing computerized physician order entry programs [40, 42, 43]. Types of medications explored for medication discrepancy were varied among the studies — for example, other than prescription medications, some studies [35, 38, 39, 44] also considered non-prescription and over-the-counter medications. Exceptionally, there was one study [36] which was specifically focused on antibiotics only. All but one study [38] clearly differentiated unintentional from intentional changes to medications from their report, or explicitly described in their methods as medication discrepancies were unintentional after clarification was sought from the medical team and/or patient. Five studies [37, 38, 42–44] evaluated both the primary and secondary outcomes. However, Schnipper et al. [40] assessed only unintentional medication discrepancies with a potential for patient harm.

**Table 1:** Characteristics of included studies

Author, Year	Country, Setting	Study design	Participant size	Target of transition	Components of intervention	Length of study	Medications assessed	Verification of discrepancy	Main results
Agrawal 2009 [35]	USA, Tertiary care academic hospital	Pre-post	19,476 patients	Admission	Multidisciplinary admission medication reconciliation, computerized reminder alert	17 ½ months	Prescription and non-prescription medications	Yes	At least 1 unintended discrepancy: 20 % (Pre) vs. 1.4 % (Post)
									Drug omission was the most common type of discrepancy in both phases
Allison 2015 [36]	USA, Academic tertiary care facility	Pre-post	200 patients	Discharge	Electronic discharge medication reconciliation, staff training	NR	Antibiotics	Yes	At least 1 antibiotic error: 23 % (Pre) vs. 11 % (Post)
									Percentage of medications with errors: 30 % (Pre) vs. 15 % (Post)
									Dosage errors were the most common type of medication error in both phases
Boockvar 2010 [37]	USA, Three academic centers	NRCT	469 patients	Nursing home to hospital transfer (admission)	Structured review	NR	Prescription medications	Yes	No difference, with and without EHR, in medication discrepancies (mean difference 0.02; 95 % CI - 0.81 to 0.85) and a high-risk discrepancies (mean difference −0.18; 95 % CI −0.22 to 0.58) per hospitalization episode, and an ADE caused by a medication discrepancy (OR 0.96; 95 % CI 0.18 to 5.01)
									46 % of prescribing discrepancies resulted in ADEs were due to drug omissions
Gimeneze- Manzorro 2011 [42]	Spain, Tertiary care hospital	Pre-post	3,781 medications	Admission	Computerized reconciliation tool integrated in a CPOE program	6 months	NR	Yes	Percentage of medications with discrepancies: 7.24 % (Pre) vs. 4.18 % (Post)
									Drug omission was the most frequent unintended discrepancy in both phases
									Omission errors: 5.8 % (Pre) vs. 3.4 % (Post)
Gimeneze- Manzorro 2015 [43]	Spain, University general hospital	Pre-post	191 patients	Admission	Nurses gather BPMH via an electronic reconciliation tool, use of CPOE	6 months	Prescription medications	Yes	At least 1 unintended discrepancy: 40.2 % (Pre) vs. 38.1 % (Post)
									Medications with unintended discrepancies: 10.6 % (Pre) vs. 6.6 % (Post)
									Of all unintended discrepancies, 144 (86.2 %) were due to drug omissions
									Omission errors: 9.2 % (Pre) vs. 5.6 % (Post)
Kramer 2007 [38]	USA, General medical unit	Pre-post	283 patients	Admission, discharge	Pharmacists and nurses collaborated to electronically complete admission and discharge medication reconciliation, discharge medication counselling	13 months	Prescription, non-prescription and herbal supplements	No	Post-implementation, patients took significantly more prescription and nonprescription medications.
Murphy 2009 [39]	USA, Academic medical center	Pre-post	SU, 149 discharges; MU, 134 discharges	Admission, discharge	Multidisciplinary MedRec using an electronic tool	2 ½ months	Prescription and non-prescription medications	Yes	Percentage of medications with unintended discrepancies: 90 % (Pre) vs. 47 % (Post) [SU]; 57 % (Pre) vs. 33 % (Post) [MU]
									On the surgical unit, omitted home medications (reduced from 21 % of orders to 0 %), omitted inpatient medications (from 8 to 1 %) and in the medical unit, omitted home and inpatient medications were both reduced from 11 to 0 %.
Schnipper 2009 [40]	USA, Two academic hospitals	RCT	322 patients	Admission, discharge	IT designed MedRec integrated into the CPOE system, interdisciplinary medication reconciliation intervention comprising novel IT and process re-design, supportive roles (e.g. training)	NR	NR	Yes	Mean number of medication discrepancies with a potential for harm per patient: 1.44 (C) vs. 1.05 (I) [RR 0.72 (0.52–0.99)]
Poole 2006 [41]	USA, Community hospital	Pre-post	100 patients	Discharge	Formation of a medication list from pre-existing electronic sources and reconciliation of discharge medications with this list	6 months	prescription medications	Yes	Statistically significant improvement with intervention vs. control in at least 1 outcome in this category; i.e., drug frequency, dose and therapeutic duplication
									Resolution of discrepancies in frequency increased by 65 %
									Resolution of discrepancies in dosages improved by 60 %
									Resolution of therapeutic duplication was addressed in 58 % of cases
Zoni 2012 [44]	Spain, University general hospital	Pre-post	162 patients	Admission	IT-designed MedRec, clinical sessions and training	12 months	Regular medications, OTC and homeopathic products	Yes	Percentage of medications with unintended discrepancies:3.5 % (Pre) vs. 1.8 % (Post)
									At least 1 unintended discrepancy: 23.7 % (Pre) vs. 14.6 % (Post)
									Drug omission was the most common unintended discrepancy
									Omission error: 2.6 % (Post) vs. 2 % (Pre)

*ADE* adverse drug event, *BPMH* best possible medication history, *CPOE* computerized physician order entry, *C* control, *EHR* electronic health record, *I* intervention, *IT* information technology, *MedRec* medication reconciliation, *MU* medical unit, *NR* not reported, *OR* odds ratio, *OTC* over-the-counter, *Pre* pre-implementation, *Post* post-implementation, *RCT* randomized controlled trial, *RR* relative risk, *SU* surgical unit

#### Quality of studies

The quality assessments of included studies were performed separately for randomized and non-randomized studies. Schnipper et al. [40] was the only randomized study assessed for its quality using the EPOC [32] risk of bias assessment tool. Except that the medication discrepancies were not assessed blindly, this study [40] was found to have a low risk of bias in terms of randomization, allocation concealment, baseline outcomes and characteristics, attrition, contamination and selection biases. The quality of non-randomized studies is described in Table 2. Using the ACROBAT-NSRI assessment tool, the overall bias among the studies were classified as moderate in five studies [36, 37, 39, 43, 44], whereas the remaining studies were judged to have a serious risk of bias.

**Table 2:** Summary of risk of bias assessment for non-randomised studies according to A Cochrane Risk of Bias Assessment Tool for Non-randomized Studies of Interventions (ACROBAT-NRSI) [33]

References	Bias due to confounding	Bias in selection of participants into the study	Bias in measurement of interventions	Bias due to departures from intended interventions	Bias due to missing data	Bias in measurement of outcomes	Bias in selection of the reported result	Overall bias
Agrawal 2009 [35]	Serious	Low	Low	No information	No information	Serious	Low	Serious
Allison 2015 [36]	Low	Low	Moderate	No information	Low	Moderate	Moderate	Moderate
Boockvar 2010 [37]	Low	Moderate	Low	Moderate	No information	Moderate	Low	Moderate
Gimeneze- Manzorro 2011 [42]	Serious	No information	Low	No information	No information	Moderate	Low	Serious
Gimeneze- Manzorro 2015 [43]	Moderate	Low	Serious	No information	Moderate	Moderate	Low	Moderate
Kramer 2007 [38]	Serious	Low	Low	Moderate	No information	Serious	Serious	Serious
Murphy 2009 [39]	No information	No information	Moderate	Moderate	No information	Moderate	Moderate	Moderate
Poole 2006 [41]	No information	Moderate	Low	Low	No information	Serious	Moderate	Serious
Zoni 2012 [44]	Low	Low	Moderate	No information	Low	Moderate	Low	Moderate

*Note*: Risk of bias judgment was based on a scale of low, moderate, serious, critical and no information

### Effectiveness of electronic MedRec interventions

Of the 10 studies that reported data on medication discrepancies, 8 studies targeting various transitions (admission, 5 studies; discharge, 1 study and multiple transitions, 2 studies) were included in the meta-analyses. Two studies [38, 41] did not contribute data in a suitable form for the meta-analysis. In one of these studies, [38] the aim was to evaluate the efficiency of an electronic tool in facilitating the reconciliation process, and did not specifically give data regarding the effectiveness of the intervention. A pharmacist-nurse initiated admission and discharge medication reconciliation by Kramer et al. [38] showed an improvement in medication history completeness after implementation of an electronic tool; that is, patients in the post-implementation group took significantly more prescription and non-prescription medications, and the total number of medications significantly exceeded the number taken by the pre-implementation group. Poole et al. [41] was the other study not included in the meta-analysis due to the outcomes evaluated. Poole et al. [41] demonstrated an effective computerization of the medication reconciliation process, and found an improvement in the safety of patients by minimizing medication discrepancies in frequency, dose and therapeutic duplication at the time of discharge — resolution of discrepancies increased by 65, 60 and 58 %, respectively.

Meta-analyses were performed in-terms of the proportion of patients with medication discrepancies, or as mean number of medication discrepancies per patient or incidence of medications with discrepancies over the total number of medications. Also, the most common type (s) of discrepancies were elaborated and synthesized quantitatively.

#### Proportion of patients with medication discrepancies

**Fig. 2:**
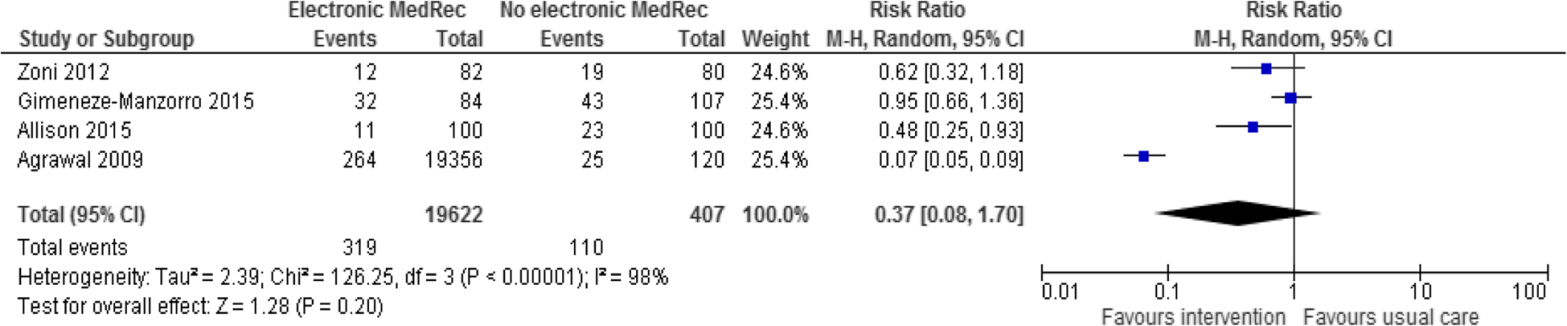
Meta-analysis of the effectiveness of electronic medication reconciliation on the proportion of patients with medication discrepancies at hospital transitions

Only four studies [35, 36, 43, 44] reported the proportion of patients with at least one medication discrepancy. Figure 2 shows the forest plot of 4 studies expressing medication discrepancies dichotomously (proportion of patients with medication discrepancies). The pooled result of such studies on this outcome showed no difference in medication discrepancies between the intervention and usual care (RR 0.37; 95 % CI 0.08 to 1.70; *p* = 0.2), and this was associated with substantial heterogeneity (*I*^*2*^ = 98 %). However, when Agrawal et al. [35] study was removed, the sensitivity analysis showed modest heterogeneity without affecting the significance difference (RR 0.70; 95 % CI 0.46 to 1.09; *p* = 0.12, *I*^*2*^ = 48 %).

#### Proportion of medications with unintended discrepancy

**Fig. 3:**
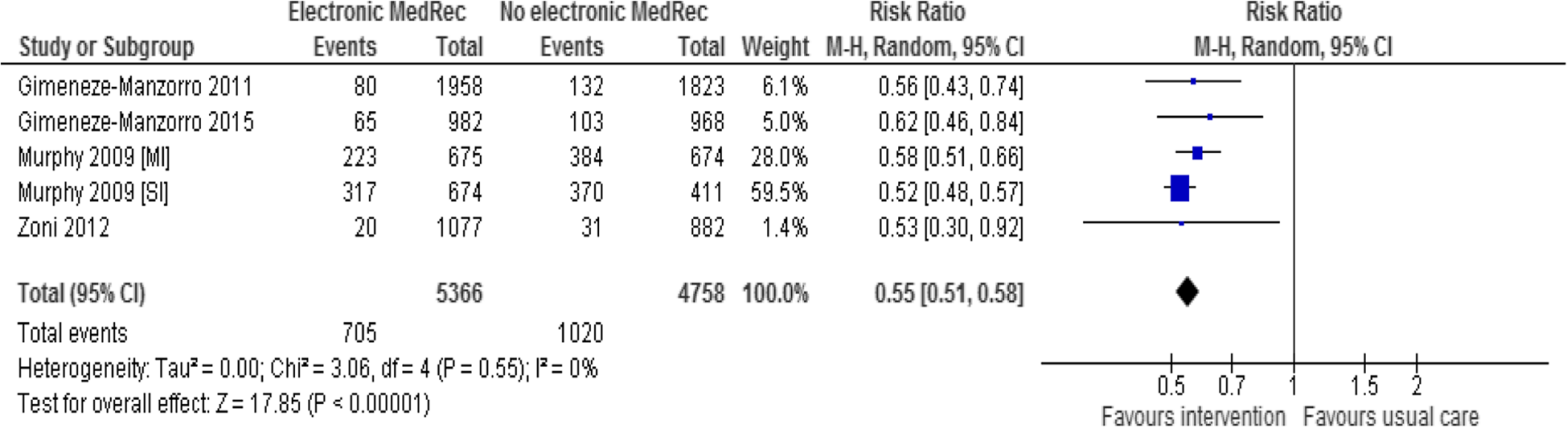
Meta-analysis of the effectiveness of electronic medication reconciliation on the incidence of medications with unintentional discrepancies over the total number of medications reconciled at hospital transitions

Four studies [39, 42–44] were able to report the incidence of unintended discrepancies over the total number of medications reconciled. One study [39] reported data for two different hospital units (surgical and medical unit) and included these data in the analysis as separate interventions. Meta-analysis of data from the five electronic medication reconciliation interventions conducted at various transitions showed a significant reduction of the incidence of medications with discrepancies in favour of the intervention (RR 0.55; 95 % CI 0.51 to 0.58; *p* < 0.00001, *I*^*2*^ = 0 %) (Fig. 3).

#### Mean medication discrepancies per patient

**Fig. 4:**
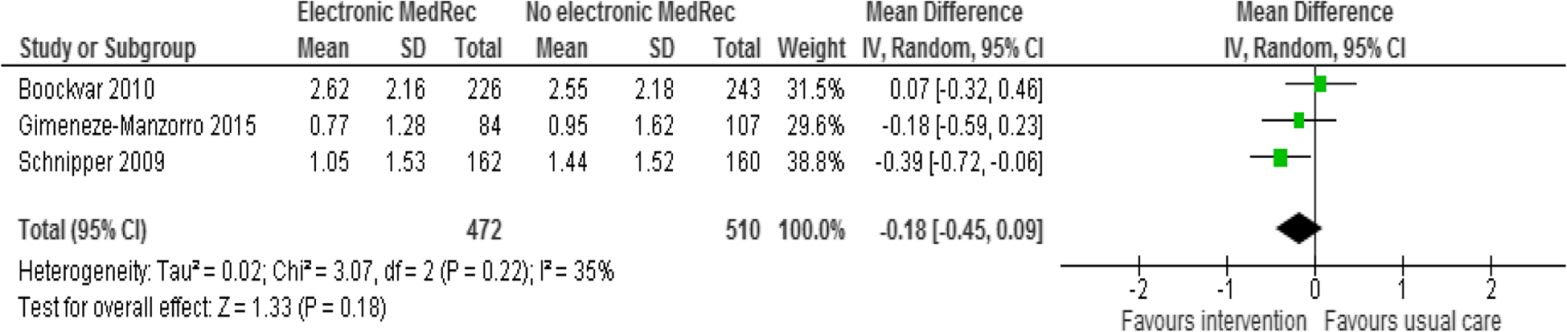
Meta-analysis of the effectiveness of electronic medication reconciliation on unintentional medication discrepancies expressed in terms of the mean number of medication discrepancies per patient

Only three studies [37, 40, 43] reported the mean number of medication discrepancies per patient as an outcome. The pooled result for these three medication reconciliation interventions did not show a significant difference between the intervention and usual care groups (mean difference −0.18; 95 % CI −0.45 to 0.09; *p* = 0.18, *I*^*2*^ = 35 %) (Fig. 4).

#### Type (s) of medication discrepancies

**Fig. 5:**
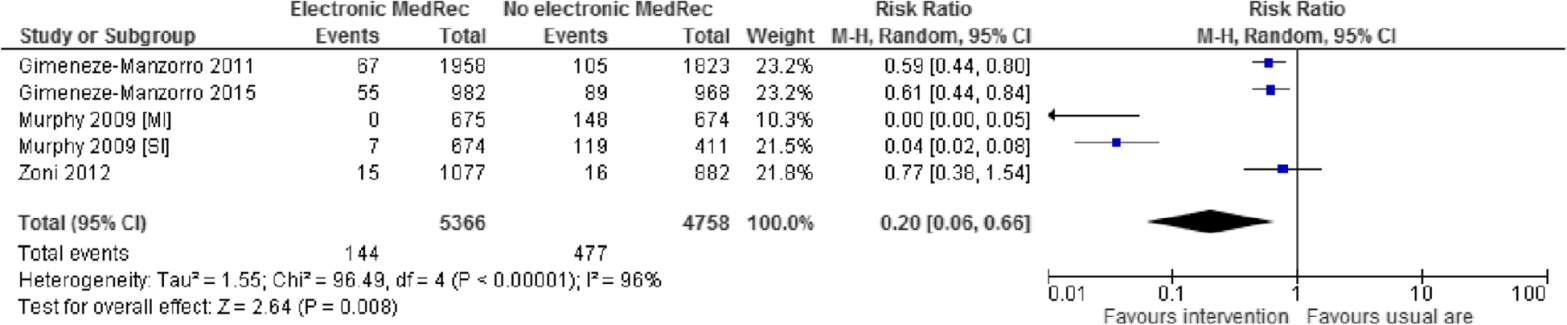
Meta-analysis of the effectiveness of electronic medication reconciliation on the percentage of omission errors over the total number of medications reconciled

Seven studies [34–36, 38, 41–43] reported the most common type (s) of medication discrepancies. Except in one study [36], the most common type of medication discrepancy identified by the majority of the studies was omission error. Four studies involving five interventions gave data in terms of the percentage of omission errors over the total number of medications reconciled, and were included in the meta-analysis. Here, Murphy et al. [39] reported data for two different hospital units; i.e., surgical and medical unit, and included in the analysis as two cohorts of interventions. Two studies [35, 36] in this meta-analysis were excluded because of an absence of a common denominator in the calculation of the pooled estimate. Meta-analysis of the five interventions expressing the proportion of omission errors over the total number of medications showed a significant reduction of 80 % in favour of the intervention (RR 0.20; 95 % CI 0.06 to 0.66; *p* = 0.008, *I*^*2*^ = 96 %) (Fig. 5). On sensitivity analysis, this effect is greatly influenced by Murphy 2009 [SI] study [39]; removal of this intervention showed a non-significant and heterogeneously distributed reduction in omission errors (RR 0.43; 95 % CI 0.17 to 1.04; *p* = 0.06, *I*^*2*^ = 91 %).

### Clinical significance of medication discrepancies

The clinical impact of medication discrepancies was reported in five studies [37, 38, 42–44]. One study [40] reported medication discrepancies with a potential for harm only. Most of the studies described that the majority of the unintended discrepancies did not cause any harm to the patient, and were grade C in severity according to the National Coordinating Council for Medication Error Reporting and Prevention (NCC MERP) classification [45]; that is, the error reached the patient but caused no harm. Two studies reported [37, 46] actual patient harm requiring intervention or prolonged hospitalization in 7 to 55 % of medication discrepancies. One study [42] compared the incidence of severe medication errors before and after implementation of an electronic tool, and found that there was a significant reduction in severity of medication discrepancies post-implementation (5.3 % vs. 2.4 %, *p* < 0.0001). Schnipper et al. [40] showed fewer medication discrepancies with the potential to cause serious harm, such as re-hospitalization in the intervention than the usual care group, 0.27 vs. 0.34 per patient, respectively. For severity assessment, four of the five studies adopted a medication error index employed by the NCC MERP [45], and evaluators in these studies were pharmacists and medical coordinators/physicians (Table 3).

**Table 3:** Clinical significance of unintentional medication discrepancies

Author, year	Tool for clinical significance evaluation	Clinical judgment determined by	Results
Boockvar 2010 [37]	NCC MERP [45]	Discussion between 2 physicians or 1 physician and 1 pharmacist	46 % of prescribing discrepancies causing ADEs were asymptomatic, 52 % were associated with symptoms and 3 % caused a prolonged or an additional hospital stay.
			No prescribing discrepancies caused permanent disability or death.
Gimeneze-Manzorro 2015 [43]	NCC MERP [45]	Consensus between the pharmacist and the medical coordinator	Grade C, 79.2 %
			Grade D, 13.6 %
			Grade E, 7.1 %
Gimeneze-Manzorro 2011 [42]	NCC MERP [45]	Pharmacist discuss with medical coordinators	Most errors were grade C in severity in both phases.
			Severe errors: Pre-implementation, 96/1,823 (5.3 %); Post-implementation, 48/1,958 (2.4 %)
Kramer 2007 [38]	Nickerson et al. 2005 [48]	NR	Pre-implementation: 3 MEs (2 category B errors, 1 category C error)
			Post-implementation: 4 MEs (3 category B errors, 1 category C error)
Zoni 2012 [44]	NCC MERP [45]	Consensus between the pharmacist and the medical coordinator	Most of the unintended discrepancies would cause no harm to the patient.
			In the pre-implementation, there were 2 patients where either patient monitoring would be required or the patient would suffer temporary damage.

*MEs* medication errors, *NCC MERP* National Coordinating Council for Medication Error Reporting and Prevention, *NR* not reported

## Discussion

### Main findings

This systematic review on electronic medication reconciliation interventions did not identify a consistent impact in minimizing the occurrence of unintentional medication discrepancies during transitions in hospital care. Specifically, pooled estimates showed a 63 % reduction in patients with medication discrepancies; however, this was not statistically significant, nor was the mean number of medication discrepancies per patient. But, the intervention had significantly reduced the percentage of medications with unintended discrepancy and drug omissions over the total number of medications reconciled. However, it should be noted that the findings were derived from a subset of studies that reported each outcome of interest. Drug omissions were the most common types of unintended discrepancies identified through an electronic tool. The clinical importance of unintended discrepancies was evaluated in five studies. There was no potentially fatal error identified, and most errors were minor in severity.

### Comparison with previous research

To the best of our knowledge, this is the first systematic review and meta-analysis that focused on the impact of electronic medication reconciliation on the rate and incidence of unintentional medication discrepancies at transitions in hospital care. Previous reviews [26, 27] evaluating the importance of medication reconciliation overall had not consistently reported the effectiveness of medication reconciliation interventions. However, latest reviews regarding medication reconciliation interventions carried out through pharmacist assessment have shown an impact on some of the clinical (e.g. all-cause readmission) and process outcomes (e.g. medication discrepancies) [46, 47]. For instance, our previous study [47] showed a substantial reduction of 66 % in patients with medication discrepancies favouring pharmacy-led interventions carried out at single transitions (either admission or discharge). However, the present study showed a non-significant reduction in either of the outcomes studied; that is, the proportion of patients with medication discrepancies, or mean number of medication discrepancies per patient. Unlike the previous review [47], the present study did not differentiate effects due to place of transition, and in that study, multiple transitions interventions were less effective in reducing medication discrepancies. In the current study, there were some studies with multiple transitions included in the meta-analyses. This might have brought differences in effect and significance.

In the present study, drug omissions were the most frequent errors and this is consistent with other published works [6, 7]. It is not surprising to observe dosage errors as the commonest errors identified in a study by Alison et al. [36]; the type of medications studied for discrepancy were antibiotics, and this group of medications are mainly indicated for acute treatment of infections. As Zoni et al. [44] allude to, there exists a relationship between chronic medicines use and the occurrence of unintended discrepancy, mainly drug omissions.

This study identified only a few of the unintended discrepancies having clinical impact on patient care. However, data from previous studies [27, 46] reported more clinically important discrepancies in 28 to 91 % of medication discrepancies. This variation might be because these reviews [27, 46] largely involved multifaceted interventions, including people and technology.

### Implications for practice and policy

While with information technology it is possible to share medication information and facilitate medical consultation between healthcare professionals, it has also resulted in reduction of medication errors and ADEs [28–30]. Most importantly, computerized physician order entry (CPOE) programs complemented with a medication reconciliation service might be an important approach in preventing the various types of medication errors occurred in a hospital setting. While a CPOE system would be able to fill the lack of prescriber’s knowledge, it would not able to detect unintentional omission of medications the patient was taking at home during transitions in hospital care [35]. It was thus, a CPOE program paired with a medication reconciliation service might be able to bridge the gaps in continuity of patient care, and further ensures a comprehensive medication history of patients. However, careful integration of the tool is very important for successful implementation of computerized medication reconciliation services. For example, Schnipper et al. [40] has depicted differences in the extent of integration of the medication reconciliation tool into a computerized provider entry applications between hospitals, and this has brought huge differences in effect. In general, effective medication reconciliation likely requires a multifaceted approach involving people, process, technology and that technology interventions alone may not consistently reduce errors.

### Strengths and limitations of the study

The main strength of this study was the exploration of the effectiveness of an electronic tool on unintentional medication discrepancies with broader inclusion criteria across a range of hospital transitions, not limited to specific transition (s). We did not select studies based on patient population (paediatric, adult) and study design. We imposed no limit on the year of publication, and we searched an extensive articles of the international literature. However, this study is not without limitations. The main limitation is that there were fewer published studies of sufficient scientific quality that adequately addressed the effects of electronic medication reconciliation on unintentional medication discrepancies. There was also heterogeneity among studies for interventions, outcomes, target of transition, study duration and methods for measuring outcomes. The number and types of medications evaluated for medication discrepancy varied among the studies. Also, the heterogeneity of the intervention needs to be considered — for example, some interventions were integrated into in an already existing computerized physician order entry programs and there were some sort of workflow redesign and staff teaching. The number and type of team who initiated an electronic interventions for medication reconciliation, and the person (s) who routinely assessed medication discrepancies were also varied. In the meta-analysis of patients with at least one discrepancy, one study of low scientific quality [35] had a great number of patients and un-proportionate sample in the intervention group and, as a result, contributed to a large extent to the pooled result and heterogeneity. The pooled estimate in this outcome did not significantly reduce the incidence of medication discrepancies and the confidence intervals crossed the line of the usual care group and were rather wide. We included only published studies in English, and the number of included studies were not enough to assess publication bias that might be arisen from language restriction and non-inclusion of non-published data.

### Implications for future study

There is a lack of high-quality studies with rigorous designs that investigate the impact of electronic medication reconciliation on medication discrepancies. Additionally, it is important that future studies should assess the clinical impact of medication discrepancies for complete evaluation of the interventions. A clear separation of intentional from unintentional medication discrepancies, and further verification of the identified discrepancies from the responsible practitioner and/or team should be noted in their report. Overall, future research should be involved at more rigorous evaluations of the interventions and evaluation of commercially available electronic medication reconciliation tools, aimed at minimizing unintentional medication discrepancies at transitions in hospital care. Studies in the paediatrics population were not identified, and studies in this regard are also urgently needed.

## Conclusion

Medication reconciliation supported by information technology was found to be an important tool for minimizing the percentage of medications with unintentional discrepancies over the total number of medications reconciled. Of particular note, omission errors were reduced in a great extent after the use of an electronic tool. But, implementation of an electronic medication reconciliation did not favour the intervention in other process outcomes; that is, patients with at least one medication discrepancy and mean number of medication discrepancies per patient. However, limitations in the available literature such as lack of well-designed studies precluded us from concluding that no effect exists. Careful integration of electronic interventions with other medication reconciliation components (i.e., supportive roles and processes) to improve outcomes of interest would be more appropriate.

## Additional files

Additional file 1: Search strategy employed in the electronic databases search (DOCX 16 kb) Additional file 2: The main reasons for exclusion of full-text articles (DOCX 19 kb)

## Abbreviations

ACROBAT-NRSI: A cochrane risk of bias assessment tool for non-randomized studies of interventions
ADE: Adverse drug event
CI: Confidence interval
CPOE: Computerized physician order entry
EPOC: Effective Practice and Organisation of Care Group
IT: Information technology
IHI: Institute for Healthcare Improvement
MedRec: Medication reconciliation
NCC MERP: National Coordinating Council for Medication Error Reporting and Prevention Scale
PRISMA: Preferred reporting items for systematic reviews and meta-analyses
RCT: Randomized controlled trial
RR: Relative risk
SD: Standard deviation
