# Supplementary material for: Impact of electronic medication reconciliation interventions on medication discrepancies at hospital transitions: a systematic review and meta-analysis
Source: BMC Med Inform Decis Mak. 2016 Aug 22;16(1):112. doi: 10.1186/s12911-016-0353-9 (PMC4994239; doi:10.1186/s12911-016-0353-9)
Supplement: Additional file 1: — Search strategy employed in the electronic databases search (DOCX 16 kb) [file 12911_2016_353_MOESM1_ESM.docx]

**Additional file 1: Search strategy employed in the electronic databases search**

**MEDLINE**

Database: Ovid MEDLINE(R) <1946 to November Week 3 2015>

1 Medication Errors/ or Medical History Taking/ or medication discrepancies.mp. or Medication Reconciliation/ (28739)

2 ((medic$ or drug$ or prescription$ or (medic$ adj2 chart$) or (medic$ adj2 record$)) adj2 review$).mp. (26100)

3 ((medic$ or drug$) adj2 histor$).mp. (67680)

4 (((medic$ adj2 chart$) or (medic$ adj2 record$)) adj2 assessment).mp. (87)

5 ((medic$ or drug$) adj2 list$).mp. (2789)

6 exp "Continuity of Patient Care"/ or electronic medication reconciliation.mp. (41625)

7 electronic health records.mp. or exp Medical Records Systems, Computerized/ or exp Electronic Health Records/ or exp Hospital Information Systems/ (46859)

8 patient admission.mp. or Patient Admission/ (20332)

9 patient discharge.mp. or Patient Discharge/ (22203)

10 inpatients.mp. or Inpatients/ (36275)

11 Patient Transfer/ or hospital transfer.mp. or Hospitalization/ (86171)

12 1 or 2 or 3 or 4 or 5 or 6 (253,018)

13 8 or 9 or 10 or 11 (152106)

14 7 and 12 and 13 (816)

15 limit 14 to (english language and humans) (688)

**PubMed**

((((((("medication errors"[MeSH Terms] OR ("medication"[All Fields] AND "errors"[All Fields]) OR "medication errors"[All Fields]) OR (("pharmaceutical preparations"[MeSH Terms] OR ("pharmaceutical"[All Fields] AND "preparations"[All Fields]) OR "pharmaceutical preparations"[All Fields] OR "medication"[All Fields]) AND discrepancies[All Fields])) OR ("medication reconciliation"[MeSH Terms] OR ("medication"[All Fields] AND "reconciliation"[All Fields]) OR "medication reconciliation"[All Fields])) OR (("pharmaceutical preparations"[MeSH Terms] OR ("pharmaceutical"[All Fields] AND "preparations"[All Fields]) OR "pharmaceutical preparations"[All Fields] OR "medication"[All Fields]) AND ("safety"[MeSH Terms] OR "safety"[All Fields]))) OR ("patient safety"[MeSH Terms] OR ("patient"[All Fields] AND "safety"[All Fields]) OR "patient safety"[All Fields])) OR (("pharmaceutical preparations"[MeSH Terms] OR ("pharmaceutical"[All Fields] AND "preparations"[All Fields]) OR "pharmaceutical preparations"[All Fields] OR "medication"[All Fields]) AND ("history"[Subheading] OR "history"[All Fields] OR "history"[MeSH Terms]))) AND (((("electronic health records"[MeSH Terms] OR ("electronic"[All Fields] AND "health"[All Fields] AND "records"[All Fields]) OR "electronic health records"[All Fields] OR ("electronic"[All Fields] AND "medical"[All Fields] AND "record"[All Fields]) OR "electronic medical record"[All Fields]) OR ("electronic health records"[MeSH Terms] OR ("electronic"[All Fields] AND "health"[All Fields] AND "records"[All Fields]) OR "electronic health records"[All Fields])) OR ("electronic prescribing"[MeSH Terms] OR ("electronic"[All Fields] AND "prescribing"[All Fields]) OR "electronic prescribing"[All Fields])) OR ("medication systems"[MeSH Terms] OR ("medication"[All Fields] AND "systems"[All Fields]) OR "medication systems"[All Fields]))) AND (((("patient admission"[MeSH Terms] OR ("patient"[All Fields] AND "admission"[All Fields]) OR "patient admission"[All Fields]) OR ("patient discharge"[MeSH Terms] OR ("patient"[All Fields] AND "discharge"[All Fields]) OR "patient discharge"[All Fields])) OR ("patient transfer"[MeSH Terms] OR ("patient"[All Fields] AND "transfer"[All Fields]) OR "patient transfer"[All Fields])) OR (("hospitals"[MeSH Terms] OR "hospitals"[All Fields] OR "hospital"[All Fields]) AND transition[All Fields])) AND (hasabstract[text] AND "humans"[MeSH Terms] AND English[lang]) [484]

**EMBASE**

Id.  Query                                                  Results 
#19. (('medication errors'/exp OR 'medication errors')            65 
     OR (medication AND discrepancies) OR 'medication
     reconciliation' OR (medication AND history) OR
     (adverse AND events) OR 'patient safety') AND
     ('electronic health records' OR 'electronic
     medical records' OR 'electronic prescribing' OR
     (medication AND record AND systems)) AND
     ('patient admission' OR 'patient discharge' OR
     'patient transfer' OR (hospital AND transition))
     AND [english]/lim AND [humans]/lim
#18. (('medication errors'/exp OR 'medication errors')            76 
     OR (medication AND discrepancies) OR 'medication
     reconciliation' OR (medication AND history) OR
     (adverse AND events) OR 'patient safety') AND
     ('electronic health records' OR 'electronic
     medical records' OR 'electronic prescribing' OR
     (medication AND record AND systems)) AND
     ('patient admission' OR 'patient discharge' OR
     'patient transfer' OR (hospital AND transition))
#17. 'patient admission' OR 'patient discharge' OR            39,746 
     'patient transfer' OR (hospital AND transition)
#16. 'electronic health records' OR 'electronic              16,447 
     medical records' OR 'electronic prescribing' OR
     (medication AND record AND systems)
#15. ('medication errors'/exp OR 'medication errors')        301,291 
     OR (medication AND discrepancies) OR 'medication
     reconciliation' OR (medication AND history) OR
     (adverse AND events) OR 'patient safety'
#14. hospital AND transition                                  36,177 
#13. 'patient transfer'                                          953 
#12. 'patient discharge'                                      1,682 
#11. 'patient admission'                                      1,070 
#10. medication AND record AND systems                        2,806 
#9.  'electronic prescribing'                                  1,915 
#8.  'electronic medical records'                              7,990 
#7.  'electronic health records'                              4,373 
#6.  'patient safety'                                        80,714 
#5.  adverse AND events                                      192,591
#4.  medication AND history                                  23,266 
#3.  'medication reconciliation'                              1,554 
#2.  medication AND discrepancies                              1,206 
#1.  'medication errors'/exp OR 'medication errors'          15,174

**CINHAL**

| **#** | **Searches** | **Results** |
| --- | --- | --- |
| S16 | S12 AND S13 AND S14 Limiters-Peer Reviewed; English Language; Abstract Available | 435 |
| S15 | S12 OR S13 OR S14 | 674 |
| S14 | S9 OR S10 OR S11 | 72,625 |
| S13 | S7 OR S8 | 61,923 |
| S12 | S1 OR S2 OR S3 OR S4 OR S5 OR S6 | 75,743 |
| S11 | (MH "Patient Admission") OR "patient admission" OR (MH "Readmission") | 13,316 |
| S10 | (MH "Transfer, Discharge") OR "patient transfer" | 3,522 |
| S9 | (MH "Inpatients") OR "hospital transition" | 58,707 |
| S8 | (MH "Electronic Order Entry")  OR "electronic prescribing" | 1,750 |
| S7 | (MH "Computerized Patient Record") OR "electronic health records" OR (MH "Medical Records+") | 60,556 |
| S6 | (MH "Patient Safety+") OR "patient safety" | 60,966 |
| S5 | (MH "Adverse Health Care Event+") OR  (MH "Adverse Drug Event+") OR  "adverse event" | 34,393 |
| S4 | (MH "Medication History") OR  "medication history" OR (MH "Patient History Taking+") | 12,227 |
| S3 | (MH "Medication Reconciliation") OR  "medication reconciliation" | 768 |
| S2 | "medication discrepancies" | 55 |
| S1 | (MH "Medication Errors+")  OR "medication errors" OR (MH "Treatment Errors+") | 14,370 |
