# Supplementary material for: Impact of electronic medication reconciliation interventions on medication discrepancies at hospital transitions: a systematic review and meta-analysis
Source: BMC Med Inform Decis Mak. 2016 Aug 22;16(1):112. doi: 10.1186/s12911-016-0353-9 (PMC4994239; doi:10.1186/s12911-016-0353-9)
Supplement: Additional file 2: — The main reasons for exclusion of full-text articles (DOCX 19 kb) [file 12911_2016_353_MOESM2_ESM.docx]

**Additional file 2: The main reasons for exclusion of full-text articles**

***Excluded studies with reasons***

Electronic prescribing tool on the impact of other medication errors

1. Abramson, E. L., et al. (2011). "Transitioning between electronic health records: Effects on ambulatory prescribing safety." Journal of General Internal Medicine 26(8): 868-874.
2. Abramson, E. L., et al. (2013). "A long-term follow-up evaluation of electronic health record prescribing safety." Journal of the American Medical Informatics Association 20(E1): e52-e58.
3. Agostini, J. V., et al. (2007). "Use of a computer-based reminder to improve sedative-hypnotic prescribing in older hospitalized patients." Journal of the American Geriatrics Society 55(1): 43-48.
4. Armada, E. R., et al. (2014). "Computerized physician order entry in the cardiac intensive care unit: Effects on prescription errors and workflow conditions." Journal of Critical Care 29(2): 188-193 186p.
5. Barron, W. M., et al. (2006). "Information technology. Implementing computerized provider order entry with an existing clinical information system." Joint Commission Journal on Quality & Patient Safety 32(9): 506-516 511p.
6. Callen, J., et al. (2010). "Accuracy of medication documentation in hospital discharge summaries: A retrospective analysis of medication transcription errors in manual and electronic discharge summaries." International Journal of Medical Informatics 79(1): 58-64.
7. Shawahna, R., et al. (2011). "Electronic prescribing reduces prescribing error in public hospitals." Journal of Clinical Nursing 20(21/22): 3233-3245 3213p.
8. Turchin, A., et al. (2011). "Unexpected effects of unintended consequences: EMR prescription discrepancies and hemorrhage in patients on warfarin." AMIA ... Annual Symposium Proceedings/AMIA Symposium 2011: 1412-1417.
9. Upperman, J. S., et al. (2005). "The impact of hospitalwide computerized physician order entry on medical errors in a pediatric hospital." J Pediatr Surg 40(1): 57-59.
10. Weant, K. A., et al. (2007). "Medication-error reporting and pharmacy resident experience during implementation of computerized prescriber order entry." Am J Health Syst Pharm 64(5): 526-530.

No control group

1. Agrawal, A., et al. (2007). "Evaluation of an electronic medication reconciliation system in inpatient setting in an acute care hospital." Studies in Health Technology & Informatics 129(Pt 2): 1027-1031.
2. Arora, V., et al. (2007). "Medication discrepancies in resident sign-outs and their potential to harm." Journal of General Internal Medicine 22(12): 1751-1755.
3. Lee, J. Y., et al. (2010). "Medication reconciliation during internal hospital transfer and impact of computerized prescriber order entry." Annals of Pharmacotherapy 44(12): 1887-1895.
4. Palchuk, M. B., et al. (2010). "An unintended consequence of electronic prescriptions: prevalence and impact of internal discrepancies." J Am Med Inform Assoc 17(4): 472-476.
5. Sinvani, L., et al. (2012). "Medication reconciliation in transition of care: Broken telephone or patient safety goal?" Journal of the American Geriatrics Society 60: S216.
6. Walke, L. M., et al. (2012). "Identification of medication discrepancies in discharge paperwork among patients in the co-operate geriatrics/surgery co management program." Journal of the American Geriatrics Society 60: S230.

Not electronic medication reconciliation

1. Bala, M., et al. (2011). "Medicines reconciliation on discharge: Implementation of a new model of working on the cardiology unit at the Leeds Teaching Hospitals NHS Trust." Clinical Pharmacist 3(4): S8.
2. Beckett, R. D., et al. (2012). "Effectiveness and feasibility of pharmacist-led admission medication reconciliation for geriatric patients." J Pharm Pract 25(2): 136-141.
3. Bergkvist, A., et al. (2009). "Improved quality in the hospital discharge summary reduces medication errors--LIMM: Landskrona Integrated Medicines Management." European Journal of Clinical Pharmacology 65(10): 1037-1046.
4. Becerra-Camargo, J., et al. (2013). "A multicentre, double-blind, randomised, controlled, parallel-group study of the effectiveness of a pharmacist-acquired medication history in an emergency department." BMC Health Serv Res 13: 337.
5. Grimes, T. C., et al. (2014). "Collaborative pharmaceutical care in an Irish hospital: uncontrolled before-after study." BMJ Qual Saf 23(7): 574-583.
6. Lee, Y. Y., et al. (2013). "Pharmacist-conducted medication reconciliation at hospital admission using information technology in Taiwan." Int J Med Inform 82(6): 522-527.
7. Lindquist, L. A., et al. (2013). "Primary care physician communication at hospital discharge reduces medication discrepancies." J Hosp Med 8(12): 672-677.
8. Lingaratnam, S., et al. (2013). "A controlled before and after study to evaluate a patient and health professional partnership model towards effective medication reconciliation." J Oncol Pharm Pract 19(1): 48-56.
9. Lu, Y., et al. (2013). "Quality improvement through implementation of discharge order reconciliation." Am J Health Syst Pharm 70(9): 815-820.
10. Schwarz, M. and R. Wyskiel (2006). "Medication reconciliation: developing and implementing a program." Critical Care Nursing Clinics of North America 18(4): 503-507.
11. Tompson, A. J., et al. (2012). "Utilizing community pharmacy dispensing records to disclose errors in hospital admission drug charts." International Journal of Clinical Pharmacology & Therapeutics 50(9): 639-646.
12. Andreoli, L., et al. (2014). "Medication reconciliation: a prospective study in an internal medicine unit." Drugs Aging 31(5): 387-393.
13. Pronovost, P., et al. (2003). "Medication reconciliation: a practical tool to reduce the risk of medication errors." Journal of Critical Care 18(4): 201-205.

Different outcome of interest

1. Al-Dorzi, H. M., et al. (2011). "Impact of computerized physician order entry (CPOE) system on the outcome of critically ill adult patients: a before-after study." BMC Medical Informatics & Decision Making 11: 71.
2. Bourne, R. S. and C. L. Choo (2012). "Pharmacist proactive medication recommendations using electronic documentation in a UK general critical care unit." Int J Clin Pharm 34(2): 351-357.
3. Cooley, T. W., et al. (2012). "Implementation of computerized prescriber order entry in four academic medical centers." Am J Health Syst Pharm 69(24): 2166-2173.
4. Ghibelli, S., et al. (2013). "Prevention of Inappropriate Prescribing in Hospitalized Older Patients Using a Computerized Prescription Support System (INTERcheck)." Drugs & Aging 30(10): 821-828 828p.
5. Gurwitz, J. H., et al. (2014). "An electronic health record-based intervention to increase follow-up office visits and decrease rehospitalization in older adults." J Am Geriatr Soc 62(5): 865-871.
6. Kirkendall, E. S., et al. (2013). "Transitioning from a computerized provider order entry and paper documentation system to an electronic health record: expectations and experiences of hospital staff." Int J Med Inform 82(11): 1037-1045.
7. Leung, A. A., et al. (2013). "Impact of vendor computerized physician order entry on patients with renal impairment in community hospitals." Journal of Hospital Medicine (Online) 8(10): 545-552.
8. Maslove, D. M., et al. (2009). "Electronic versus dictated hospital discharge summaries: a randomized controlled trial." Journal of General Internal Medicine 24(9): 995-1001
9. McCoy, A. B., et al. (2015). "Clinician satisfaction before and after transition from a basic to a comprehensive electronic health record." Journal of Investigative Medicine 63(2): 467.
10. Mekhjian, H. S., et al. (2002). "Immediate benefits realized following implementation of physician order entry at an academic medical center." J Am Med Inform Assoc 9(5): 529-539.
11. Moy, N. Y., et al. (2014). "Development and sustainability of an inpatient-to-outpatient discharge handoff tool: a quality improvement project." Jt Comm J Qual Patient Saf 40(5): 219-227.
12. Munck, L. K., et al. (2014). "The use of shared medication record as part of medication reconciliation at hospital admission is feasible." Danish Medical Journal 61(5): A4817.
13. Palma, J. P., et al. (2011). "Impact of electronic medical record integration of a handoff tool on sign-out in a newborn intensive care unit." Journal of Perinatology 31(5): 311-317 317p.
14. Patterson, M. E., et al. (2014). "Comprehensive electronic medical record implementation levels not associated with 30-day all-cause readmissions within Medicare beneficiaries with heart failure." Appl Clin Inform 5(3): 670-684.
15. Pinto Thirukumaran, C., et al. (2015). "The impact of electronic health record implementation and use on performance of the Surgical Care Improvement Project measures." Health Serv Res 50(1): 273-289.
16. Schnipper, J. L., et al. (2011). "Development of a tool within the electronic medical record to facilitate medication reconciliation after hospital discharge." Journal of the American Medical Informatics Association 18(3): 309-313.
17. Stengel, D., et al. (2004). "Comparison of handheld computer-assisted and conventional paper chart documentation of medical records: a randomized, controlled trial." Journal of Bone & Joint Surgery, American Volume 86-A(3): 553-560 558p.
18. Turchin, A., et al. (2007). "The use of electronic medication reconciliation to establish the predictors of validity of computerized medication records." Studies in Health Technology & Informatics 129(Pt 2): 1022-1026.
19. Showalter, J. W., et al. (2011). "Effect of standardized electronic discharge instructions on post-discharge hospital utilization." J Gen Intern Med 26(7): 718-723.
20. Phansalkar, S., et al. (2015). "Impact of incorporating pharmacy claims data into electronic medication reconciliation." American Journal of Health-System Pharmacy 72(3): 212-217 216p.
21. Moore, P., et al. (2011). "Medicines reconciliation using a shared electronic health care record." Journal of patient safety 7(3): 148-154.

Review

1. Bayoumi, I., et al. (2009). "Interventions to improve medication reconciliation in primary care." Ann Pharmacother 43(10): 1667-1675.
2. Motamedi, S. M., et al. (2011). "The efficacy of computer-enabled discharge communication interventions: a systematic review." BMJ Quality & Safety 20(5): 403-415
3. Niazkhani, Z., et al. (2009). "The impact of computerized provider order entry systems on inpatient clinical workflow: a literature review." Journal of the American Medical Informatics Association 16(4): 539-549 511p.
4. Reckmann, M. H., et al. (2009). "Does computerized provider order entry reduce prescribing errors for hospital inpatients? A systematic review." Journal of the American Medical Informatics Association 16(5): 613-623 611p
5. van Rosse, F., et al. (2009). "The effect of computerized physician order entry on medication prescription errors and clinical outcome in pediatric and intensive care: a systematic review." Pediatrics 123(4): 1184-1190 1187p.

Study protocol

1. Okoniewska, B. M., et al. (2012). "The Seamless Transfer-of-Care Protocol: a randomized controlled trial assessing the efficacy of an electronic transfer-of-care communication tool." BMC Health Serv Res 12: 414.

Not hospital-based

1. Shivji, F. S., et al. (2015). "Improving communication with primary care to ensure patient safety post-hospital discharge." British Journal of Hospital Medicine 76(1): 46-49.
